# Multimodality imaging evaluation of primary testicular extranodal natural killer/T-cell lymphoma: two case reports

**DOI:** 10.3389/fmed.2023.1183564

**Published:** 2023-05-30

**Authors:** Wenpeng Huang, Xiaonan Liu, Liming Li, Yongbai Zhang, Yuan Gao, Jianbo Gao, Lei Kang

**Affiliations:** ^1^Department of Nuclear Medicine, Peking University First Hospital, Beijing, China; ^2^Department of Pathology, The First Affiliated Hospital of Zhengzhou University, Zhengzhou, Henan, China; ^3^Department of Radiology, The First Affiliated Hospital of Zhengzhou University, Zhengzhou, Henan, China

**Keywords:** natural killer/T-cell lymphoma, testis, computed tomography, ^18^F-FDG PET/CT, case report, magnetic resonance imaging

## Abstract

**Background:**

Extranodal natural killer/T-cell lymphoma (ENKTCL) is a distinct pathological entity and accounts for ~10% of T-cell lymphomas. The histological features of ENKTCL include angiodestruction and coagulative necrosis and the association with EBV infection. ENKTCL is typically aggressive and mainly affects the nasal cavity and nasopharyngeal region. However, some patients can present with distant nodal or extranodal involvement such as the Waldeyer ring, gastrointestinal tract, genitourinary organs, lung, thyroid, skin, and testes. Compared to ENKTCL of nasal type, primary testicular ENKTCL is very rare and has a lower age of onset and faster clinical progression, with tumor cell dissemination occurring early in the disease.

**Case report:**

Case 1: A 23-year-old man presented with 1 month of right testicular pain and swelling. Enhancement CT revealed increased density in the right testis, uneven increased enhancement, discontinuity of the local envelope, and multiple trophoblastic vessels in the arterial phase. Testicular ENKTCL was diagnosed by post-operative pathology. The patient underwent a follow-up ^18^F-FDG PET/CT imaging 1 month later and found elevated metabolism in the bilateral nasal, left testicular, and right inguinal lymph nodes. Unfortunately, the patient received no further treatment and died 6 months later. Case 2: A 2-year-old male child presented with an enlarged right testicle, MRI showed a mass in the right epididymis and testicular area, which showed low signal on T1WI, high signal on T2WI and DWI, and low signal on ADC. Meanwhile, CT showed soft tissue in the lower lobe of the left lung and multiple high-density nodules of varying sizes in both lungs. Based on the post-operative pathology, the lesion was diagnosed with primary testicular ENKTCL. The pulmonary lesion was diagnosed as hemophagocytic lymphohistiocytosis associated with EBV infection. The child was given SMILE chemotherapy, but pancreatitis was induced during chemotherapy, then he died 5 months later after chemotherapy.

**Conclusion:**

Primary testicular ENKTCL is very rare in clinical practice, typically presenting as a painful testicular mass, which can mimic inflammatory lesions and cause diagnostic challenges. ^18^F-FDG PET/CT plays pivotal roles in the diagnosis, staging, evaluation of treatment outcomes and prognosis evaluation in patients with testicular ENKTCL, and it is helpful to assist clinical practice to better formulate individualized treatment plans.

## Introduction

Lymphomas can develop anywhere in the body, both inside the lymphatic system (nodal) and outside (extranodal), accounting for about 4% of adult cancer (1). Approximately 90% of lymphomas are non-Hodgkin lymphoma, which can be divided into more than 70 disease entities based on histopathology, immunophenotypics, genetics, and clinical manifestations. According to the World Health Organization's (WHO) classification of lymphoid tumors, extranodal natural killer/T-cell lymphoma (ENKTCL) is classified as a distinct pathological entity (2). ENKTCL constitutes ~10% of T-cell lymphomas and exhibits a higher prevalence in Asia, Central, and South America compared to Western nations (3–5). The histological features of ENKTCL include angiodestruction and coagulative necrosis which are associated with EBV infection (6). ENKTCL is aggressive and mainly affects the nasal cavity and nasopharyngeal region, and this particular histopathologic type is therefore known as ENKTCL, nasal type. In some cases, patients may exhibit distant nodal or extranodal involvement, involving sites such as the Waldeyer ring, gastrointestinal tract, lung, thyroid, skin, testis, or adrenal glands (7–9). The incidence of relapse, refractory disease, and mortality associated with ENKTCL is notably elevated (10).

Here, we present two unique cases of primary testicular NK/T-cell lymphoma, one of which was characterized by bilateral nasal, left testicular, and right inguinal lymph node involvement following surgery, and the other associated with hemophagocytic lymphohistiocytosis. Both cases exhibited a dismal clinical course and prognosis. In addition, we summarized the ^18^F-FDG PET/CT findings of testicular NK/T-cell lymphoma from the literature in Table 1.

**Table 1:** ^18^F-FDG PET/CT manifestations of primary testicular NK/T-cell lymphoma.

**No**.	**References**	**Age**	**Clinical presentation**	**Primary Sites**	**Max diameter/cm**	**SUVmax**	**Involved organs**	**Management**	**Prognosis**
1	Hallak et al. (5)	35	Right testicular mass	Right testis	5.8	8.9 (sinonasal lesion)	Sinonasal relapses	Surgery + chemotherapy +RT	Alive at 31 mo
2	Shu et al. (7)	39	Painful left testicular mass and night sweats	Left testis	NA	9.5	NA	NA	NA
3	Naboush et al. (10)	38	Nasal granuloma, dysphagia, and multiple oral ulcers.	Right testis	NA	NA	Sinuses and bone marrow	Surgery + chemotherapy	Died of fulminant hepatitis B infection
4	Tombet et al. (24)	28	Right nasal swelling, left scrotal swelling, fever, night sweat, and weight loss	Left testis	7.4	NA	Right maxillary sinus	Surgery + chemotherapy	Died at 1 mo

## Case presentation

### Case 1

**Figure 1:**
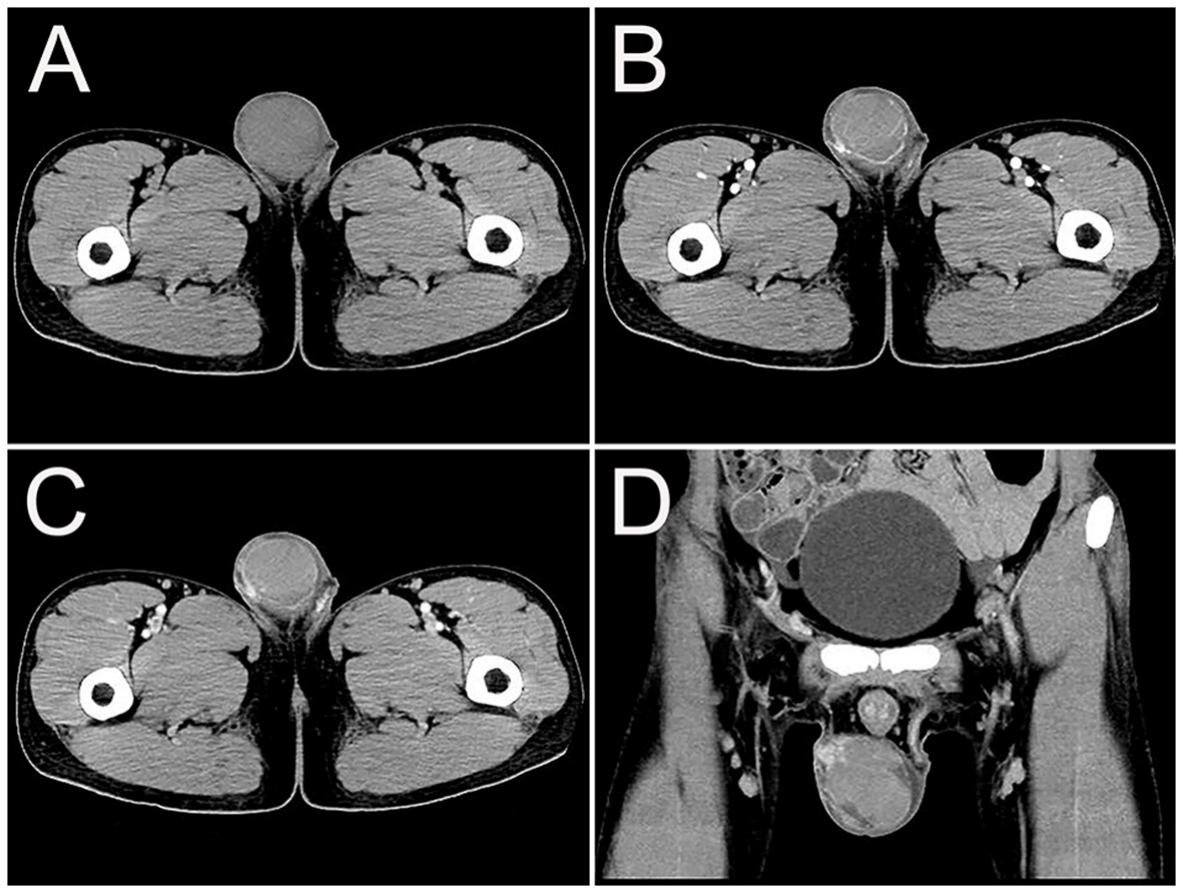
Computed tomography (CT) images of primary right testicular NK/T-cell lymphoma. **(A)** The transverse image showed a mass with increased density (32 HU) in the right testis. **(B)** The arterial phase transverse image showed patchy increased enhancement (54 HU), and multiple trophoblastic vessels in the tumor. **(C, D)** Venous phase transverse image and coronal images showed uneven moderate enhancement of tumor (66 HU), interrupted continuity of the local envelope.

A 23-year-old man presented with 1 month of right testicular pain and swelling. The symptoms further worsened and he came to the hospital for further examination. Physical examination revealed that the right testicle was significantly enlarged, hard, and painful to touch. Laboratory tests revealed C-reactive protein 15.39 mg/L, lactate dehydrogenase 1,050 U/L, Epstein-Barr virus (EBV) DNA 10.52 × 10^2^ copies/ml, CA125 76.60 U/mL, CA15-3 14.06 U/mL, Alpha-fetoprotein (AFP) 4.91 ng/ml, and carcinoembryonic antigen (CEA) 7.19 ng/mL. The patient was previously healthy without testicular enlargement and had no family history of hereditary disease. The patient underwent an ultrasound, which showed an enlarged right testicle (6.2 cm × 3.5 cm × 4.7 cm) with hypoechoic parenchyma. Color Doppler showed the presence of abundant internal blood flow signals. Further enhancement computed tomography (CT) examination revealed a mass with increased density (32 HU) in the right testis (Figure 1A). The mass showed patchy increased enhancement, interrupted continuity of the local envelope, and multiple trophoblastic vessels in the arterial phase (Figure 1B). The CT attenuation values in the arterial and venous phases were 54 and 66 HU, respectively (Figures 1C, D). The initial diagnosis was considered to be seminoma by CT, but the final diagnosis of extranodal NK/T-cell lymphoma (ENKTCL) was made by pathological biopsy. The patient underwent a craniocerebral MRI examination, which revealed no abnormalities and ruled out a lesion in the nasal cavity.

**Figure 2:**
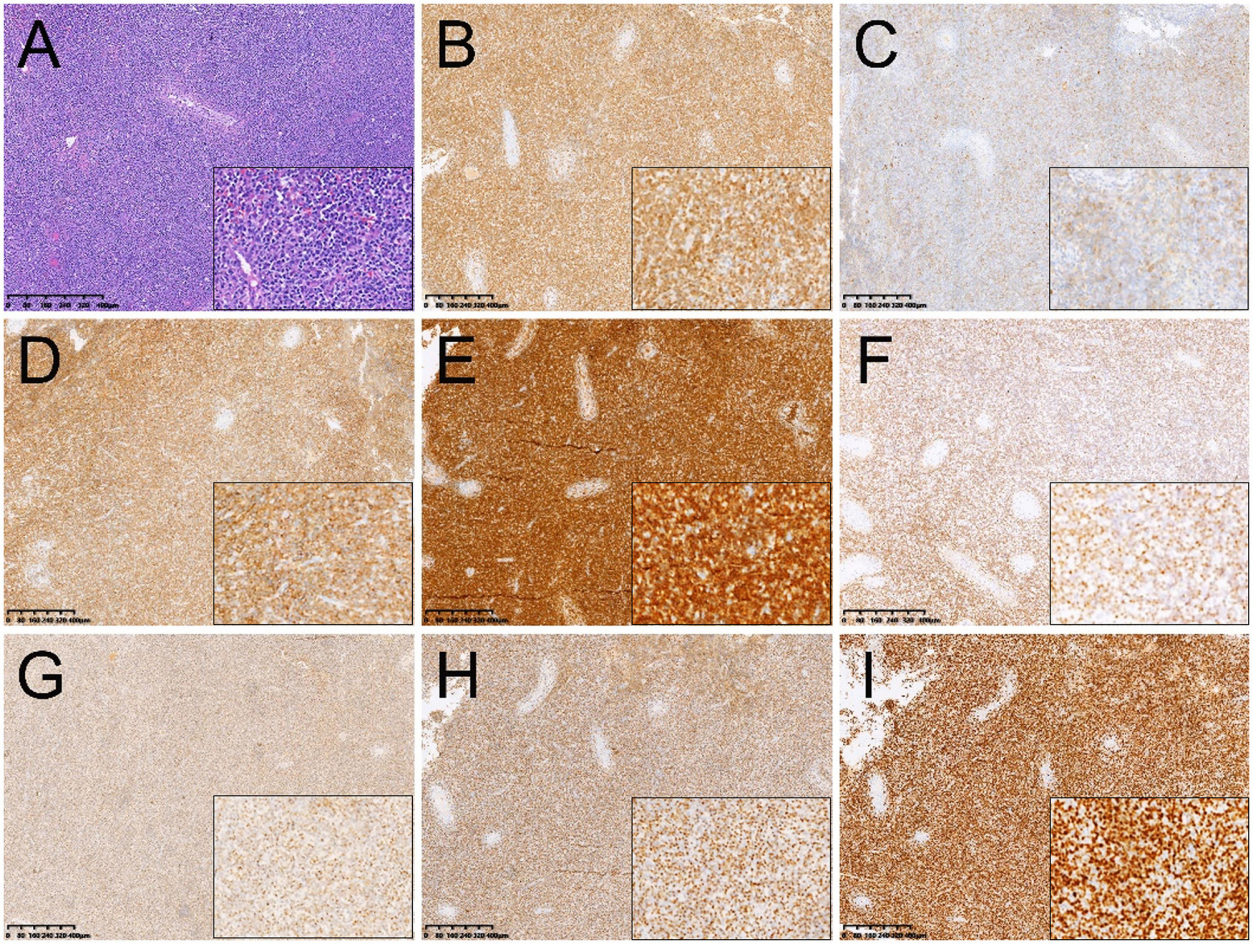
Histopathological and immunohistochemical images. **(A)** Hematoxylin-eosin (HE) staining (magnification ×40 & 200) showed a large number of diffusely infiltrating tumor cells among the germinal tubules. The cells with abundant cytoplasm showed different sizes, large deep-stained nuclei, and easily visible nuclear schizophrenia. Immunohistochemistry showed that the short spindle cells were positive for CD3 **(B)**, CD20 **(C)**, CD43 **(D)**, CD56 **(E)**, EBER **(F)**, granzyme B **(G)**, TIA-1 **(H)**. Ki-67 was observed to be positive in 80% of the tumor cells **(I)** (magnification ×40 & 200).

The patient then underwent right orchiectomy, during which the medium-hard right testicle mass was found to be ~8.0 cm × 5.0 cm × 3.5 cm in size, with a grayish-red cut surface. Post-operative pathology revealed that the basic structure of the testis was destroyed. There were a large number of diffusely infiltrating tumor cells among the germinal tubules. The cells with abundant cytoplasm showed different sizes, large deep-stained nuclei, and easily visible nuclear schizophrenia. Coagulative necrosis was found inside with inflammatory cell infiltration around the necrotic area (Figure 2A). Immunohistochemistry showed positive expression of EBER, CD3, CD30, CD20, CD43, CD56, T-cell intracellular antigen 1 (TIA-1), granzyme B (Figures 2B–H). Ki-67 was observed to be positive in 80% of the tumor cells (Figure 2I).

**Figure 3:**
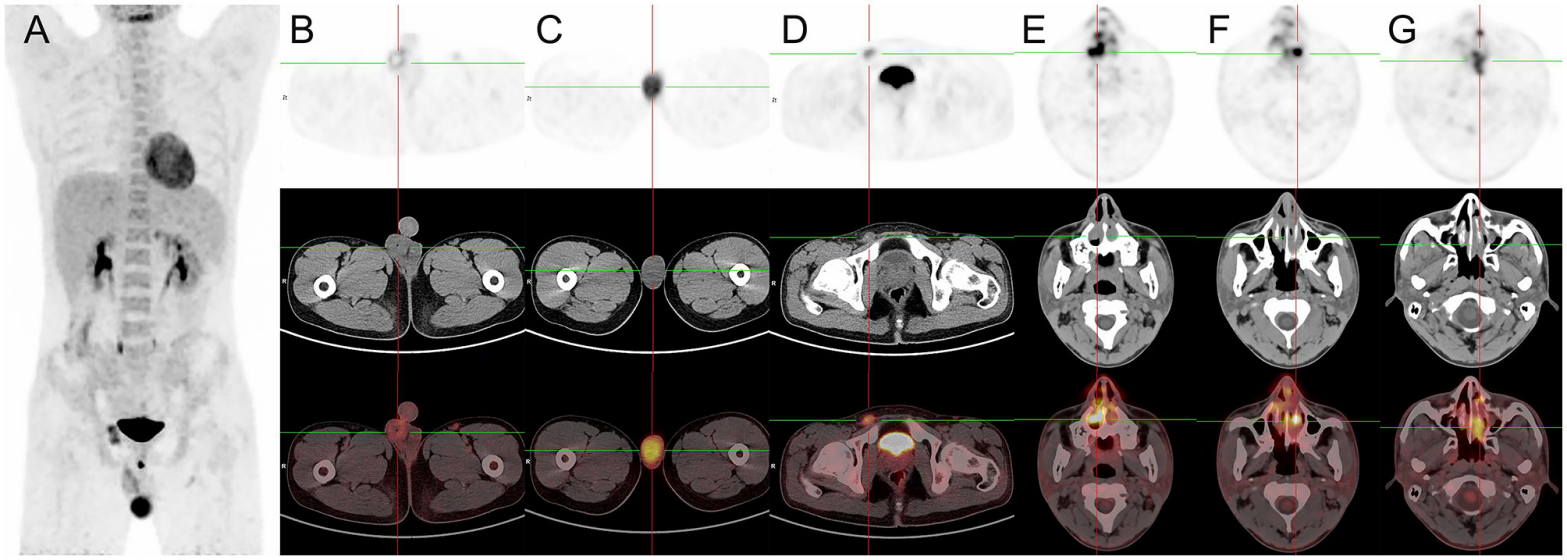
^18^F-FDG PET/CT images of primary right testicular NK/T-cell lymphoma. **(A)** The anteroposterior 3-dimensional maximum intensity projection image (MIP) demonstrated elevated metabolism in the left testicular, and right inguinal lymph nodes. **(B)** The transverse images showed a missing right testis and FDG-avid circular soft tissue in the right scrotum (SUVmax = 3.9), relating to post-operative changes. **(C)** The transverse images showed the left testicle was enlarged at a maximum dimension of about 3.9 cm × 6.0 cm and had a significantly high FDG uptake (SUVmax = 9.7). **(D)** The transverse images showed a swelling right inguinal lymph node (1.3 cm × 2.1 cm) had an increased FDG uptake (SUVmax = 6.8). **(E–G)** The transverse images showed a soft tissue mass with unevenly increased FDG uptake found in the bilateral nasal cavity, inferior turbinates, and left septal sinus (SUVmax = 24.5), suggesting lymphoma involvement.

The patient underwent a follow-up ^18^F-FDG PET/CT scan 1 month later, which revealed a missing right testis and FDG-avid circular soft tissue in the right scrotum (SUVmax = 3.9), relating to post-operative changes. However, the left testicle was enlarged at a maximum dimension of about 3.9 cm × 6.0 cm and had a significantly high FDG uptake (SUVmax = 9.7). A swelling right inguinal lymph node (1.3 cm × 2.1 cm) had an increased FDG uptake (SUVmax = 6.8). Meanwhile, a soft tissue mass with unevenly increased FDG uptake was found in the bilateral nasal cavity, inferior turbinates, and left septal sinus (SUVmax = 24.5), suggesting lymphoma involvement (Figure 3). The patient had a clinical Ann Arbor stage IV and an International Prognostic Index (IPI) score of 3. Unfortunately, the patient and his family chose to give up receiving further treatment because of the financial reasons and died 6 months later.

### Case 2

**Figure 4:**
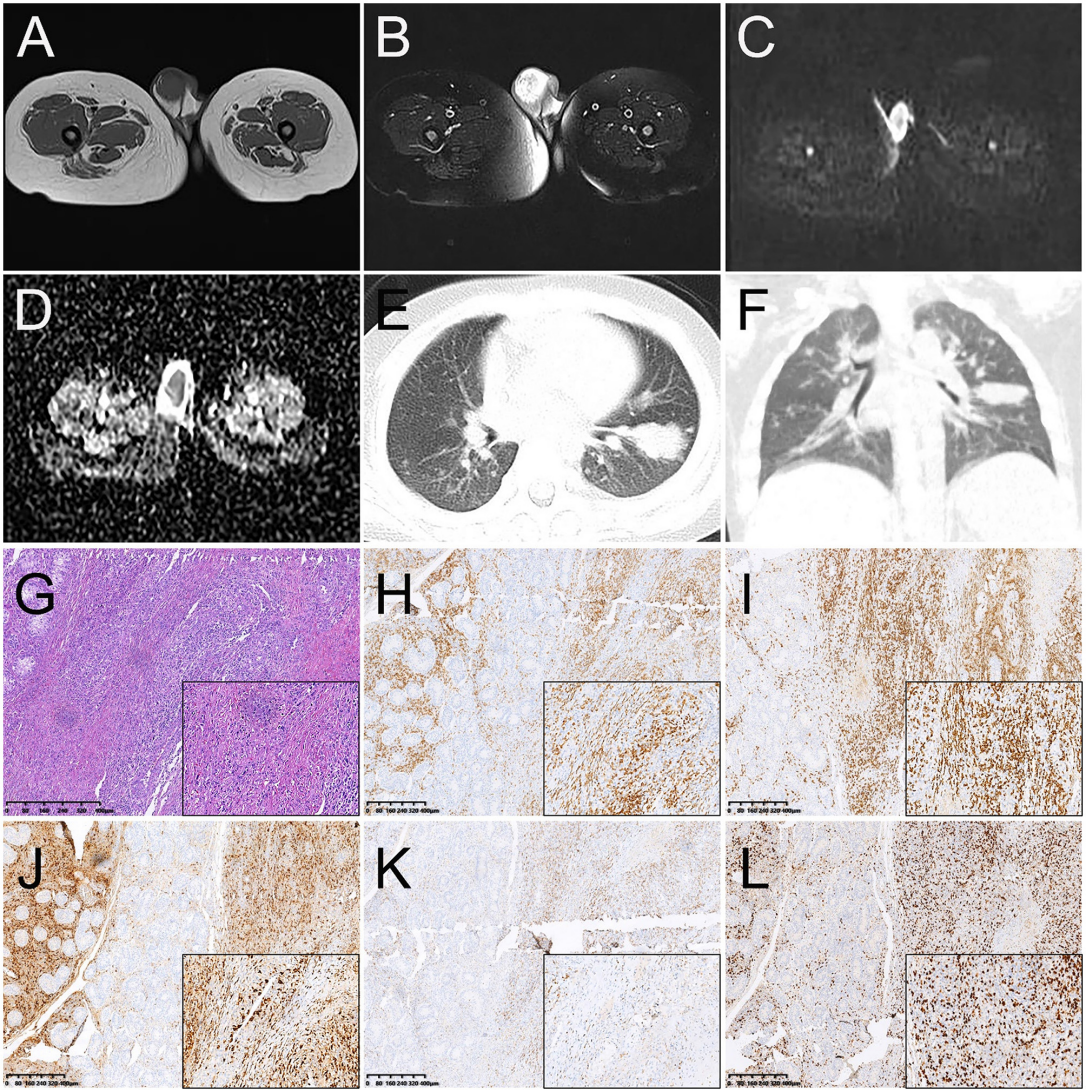
Magnetic resonance images (MRI) and CT images of primary right testicular NK/T-cell lymphoma **(A–F)**. **(A)** T1WI transverse image shows an increase in the size of the right testicle with a heterogeneous low signal lesion. **(B)** T2WI transverse image shows a heterogeneous high signal lesion. The lesions showed a high signal on DWI **(C)** and a low signal on ADC images **(D)**. **(E, F)** The transverse image and coronal images of the lung window on CT showed multiple high-density nodules in different sizes in both lungs with the largest one at 3.0 cm × 1.8 cm in size in the lower lobe of the left lung, suggesting lung metastasis. Histopathological and immunohistochemical images **(G–L)**. **(G)** Hematoxylin-eosin (HE) staining (magnification ×40 & 200) showed diffuse infiltration of tumor cells into the testicular parenchyma and diffuse growth of lymph-like cells between the germinal tubules with varying sizes and nuclear schizophrenia. Immunohistochemistry showed that the short spindle cells were positive for CD3 **(H)**, CD8 **(I)**, granzyme B **(J)**, and TIA-1 **(K)**. Ki-67 was observed to be positive in 70% of the tumor cells **(L)** (magnification ×40 & 200).

A 2-year-old male child was found with an enlarged right testicle 10 days ago. In physical examination at the hospital, a mass of about 2 cm in diameter in the right scrotal area of the child was palpated with clear borders, pressure pain, and no obvious stalk tip into the abdominal cavity. The size of the mass did not decrease after squeezing and the transillumination test was negative. Laboratory tests revealed AFP 1.91 ng/ml, lactate dehydrogenase of 295 U/L, EBV DNA 8.48 × 10^3^ copies/mL, positive IgG antibody to EBV capsid antigen, and positive IgG antibody to EBV nuclear antigen. The child was previously healthy with no testicular enlargement and no family history of hereditary disease. The ultrasound revealed the swelling right testicle with an internal hypoechoic zone, which did not exclude inflammatory lesions. MRI examination found that a mass-like abnormal signal was seen in the right epididymis and testicular area. The lesion at about 2.9 cm × 2.0 cm × 2.4 cm showed low signal on T1WI, high signal on T2WI and DWI, and low signal on ADC. While an abnormal signal was seen in the right epididymal area (Figures 4A–D). The shape and signal of the left testis showed no significant abnormalities. CT examination further showed multiple high-density nodules of different sizes in both lungs with the largest one at 3.0 cm × 1.8 cm in size in the lower lobe of the left lung, suggesting lung metastasis (Figures 4E, F).

After the consent of the child's parents, the right orchiectomy was performed. During surgery, the right testicle was found to be about 3.6 cm × 2.6 cm × 1.8 cm in size, and a grayish-red medium-hard mass was found inside the testicular parenchyma with a size of about 2.1 cm × 1.5 cm × 1.2 cm. Post-operative pathology revealed diffuse infiltration of tumor cells into the testicular parenchyma and diffuse growth of lymph-like cells between the germinal tubules with varying sizes and nuclear schizophrenia. Necrosis can also be partly seen and atrophy of the residual testicular tissue was found (Figure 4G). Immunohistochemistry showed positive expression of CK, CD3, CD8, TIA-1, CD30 (focal), EMA (focal), and granzyme B (Figures 4H–K), but the negative expression of CD20, CD5, CD4, and EBNA2. Ki67 was observed to be positive in 70% of the tumor cells (Figure 4L). In addition, an Epstein-Barr virus-encoded RNA (EBER) assay was performed and showed positive *in situ* hybridization for EBER. Lymphocyte immunoassay showed an increased percentage by 24.63% of NK cells (normal range, 7.0–14.0%). A cranial MRI revealed no abnormalities and ruled out a lesion in the nasal cavity. Based on the information on immunophenotype, molecular detection, and laboratory test results, the patient was diagnosed with primary testicular ENKTCL with hemophagocytic lymphohistiocytosis (HLH) associated with EBV infection in the lungs. The patient had a clinical Ann Arbor stage I and an IPI score of 3.

After the exclusion of contraindications to chemotherapy, the child was given a SMILE chemotherapy regimen (DXM + MTX + IFO + L-Asp + VP-16). During chemotherapy, the child developed abdominal pain, and elevated blood and urine amylase. Ultrasound displayed an enlarged pancreas with dilated pancreatic ducts so pancreatitis was considered and chemotherapy was suspended. The child died 5 months after leaving the hospital.

## Discussion

Most lymphomas in the testicular region are disseminated from extra-testicular lymphomas. In primary testicular lymphoma (PTL), the testis is the only involved site at presentation, with no involvement of other nodes or organs (11). It is a hematologic tumor with high malignancy, accounting for 1–7% of testicular tumors, and is most likely to occur in men over 60 years of age (12). The most common pathological type of PTL is diffuse large B-cell lymphoma (DLBCL) (80–98%), while ENKTCL is rare (12, 13). Among the two subtypes of ENKTCL, the more prevalent subtype, known as the nasal type, typically involves midline structures such as the nasal cavity, nasopharynx, and sinuses. Conversely, the other subtype, which is rarer, can manifest in diverse locations such as the skin, testes, liver, gastrointestinal tract, lungs, orbits, salivary glands, and muscles (7–9, 14, 15), or may present as a disseminated form of the disease, known as NK/T cell leukemia (16).

Compared to ENKTCL of nasal type, primary testicular ENKTCL is much less reported and has a lower age of onset and rapid clinical progression. Our reports about one adolescent case and one infant case are consistent with previous reports. It is important to measure the circulating EBV DNA as a surrogate biomarker of lymphoma load (17). The child had concomitant EBV infection associated with hemophagocytic lymphohistiocytosis and his EBV-DNA load was elevated, supporting the diagnosis. In contrast to ENKTCL affecting other sites, primary testicular ENKTCL is frequently diagnosed at Ann Arbor stage I, likely due to the early detection of symptoms and timely diagnosis facilitated by the testis' accessible location at the surface of the body.

The pathological findings of primary testicular ENKTCL consist of atypical lymphoid cells, inflammatory cells, and eosinophils, that surround and infiltrate the seminiferous tubules, accompanied by angiocentricity and angiodestruction (18, 19). ENKTCL also exhibits complex molecular genetic changes. Based on terminal repeat sequence analysis, EBV is clonal in NK/T cells and exists as an episomal virus that cannot be integrated into the host genome (20–23). Lymphoma cells exhibit the typical immunophenotype of CD2+, CD56+, cytoplasmic CD3ε+, surface CD3–, and cytotoxic molecules (granzyme B, perforin, TIA1)+ (20). Expression of CD30, a cytokine receptor that induces viral infection in T and B cells, is associated with a poorer prognosis in patients with ENKTCL (15). Primary testicular NK/T-cell lymphoma tends to involve the central nervous system, nasopharynx, skin, lymph nodes, gastrointestinal tract, spleen, and contralateral testis, which may be associated with the expression of abundant CD56 molecules in these sites (24, 25). The testicular NK/T-cell lymphomas described in our study exhibit characteristic features, characterized by densely packed and widely disseminated populations of medium to large atypical lymphocytes, and are known to demonstrate EBV positivity.

Known for its aggressive clinical course, if the primary tumor is not detected in the early stage, ENKTCL may spread to many organ systems and affect many organs or systems (1). CT and MRI have been widely used to diagnose nasal ENKTL, but for non-nasal origin, CT and MRI cannot be used for staging because occult involvement of the nose and other anatomical sites cannot be detected. In this case, ^18^F-FDG PET/CT is performed to accurately detect lesions that are not detected in conventional imaging methods and to clarify the location and number of abnormal metabolic lesions (8, 26, 27). A study conducted by Fujiwara et al. (28) showed that ^18^F-FDG PET/CT was superior to conventional methods (enhanced CT, biopsies from primary sites, and bone marrow examinations) in detecting nodal lesions (100 vs. 93%), extranodal lesions (94 vs. 61%) and cutaneous lesions (100 vs. 65%). Similar findings have been found in a study made by Liu et al. (29). In their study of 39 patients with cutaneous ENKTCL, ^18^F-FDG PET/CT had high accuracy in the diagnosis of cutaneous and extracutaneous lesions in patients with ENKTCL, detecting 48 cutaneous and 88 extracutaneous regions while conventional methods detected only 34 cutaneous lesions and 61 extracutaneous lesions, with statistically significant differences in detection rates. Filizoglu et al. (8) reported a unique case of ENKTCL with widespread cutaneous and subcutaneous involvement. Huang et al. (30) reported a case of ENKTCL involving the left vocal cord, which showed focal FDG uptake on PET/CT. All of the above highlight the value of ^18^F-FDG-PET/CT in ENKTCL diagnosis and staging. Regional lymph node involvement has been reported as a rare event, even in patients with primary testicular ENKTCL patients with the widely involved disease (9). However, the first case presented with right inguinal lymph node involvement, and the possibility of lymph node involvement still needs to be considered in primary testicular ENKTCL patients. Primary testicular ENKTCL should be differentiated from seminoma, yolk sac tumor, and testicular inflammation. Testicular seminomatous cell tumor mostly occurs in patients aged 30–40 years old, often with a history of cryptorchidism. The tumor has an equal signal to normal testis on T1WI with clear margins and low signal on T2WI. After enhancement, the solid part is mildly enhanced and the fibrovascular part is highly enhanced. It may be accompanied by syringomyelia and lymph node metastasis. Yolk cystic tumors are most often seen in children, and laboratory tests tend to reveal increased AFP. Mostly equal or slightly low signal on T1WI and slightly high signal on T2WI, which may be accompanied by hemorrhage. Testicular inflammation often has symptoms of infection such as fever or pain, usually secondary to urinary tract infections, and imaging manifests as diffuse enlargement of the testicles, which might be accompanied by syringomyelia.

^18^F-FDG PET/CT is also valuable for the prognosis of ENKTCL and can also be used as a baseline for comparing interim and end-of-treatment scans, helping to guide the clinic to appropriate and effective treatment modalities and protocols (29, 31–39). Bai et al. (40) retrospectively analyzed the effect of SUVmax on the survival of ENKTCL and showed that SUVmax was an independent prognostic factor for overall survival (OS). A multicenter retrospective study conducted by Pak et al. (41) included 36 patients with ENKTCL to assess the prognostic value of pre-treatment PET/CT metabolic parameters, the results showed that total lesion glycolysis (TLG) was the only significant predictor of progression-free survival (PFS).

Currently, the International Prognostic Index (IPI) and the Korean Prognostic Index (KPI) are the most common prognostic models (42, 43). However, these prognostic scoring systems have been reported to have limited predictive accuracy for ENKTCL (44, 45). Li et al. (46) developed nomograms using pretreatment ^18^F-FDG PET/CT parameters and clinical parameters and found that PET/CT could be used as an effective tool for individualized prediction of PFS and OS in 171 ENKTCL patients. The nomograms had better predictive accuracies with a C-index of 0.729 and 0.736 for PFS and OS, respectively, compared with IPI and KPI. Guo et al. (47) found that deep learning analysis with ^18^F-FDG PET/CT provides an effective method for predicting the prognosis of ENKTCL patients. The identified feature prediction similarity index and maps may potentially contribute to patient stratification in treatment. Wang et al. (48) developed a PET radiomics-based model to predict PFS and OS in 110 nasal-type ENKTCL, and the R-signature constructed in the training and validation cohorts had moderate predictive ability (AUC = 0.788 and 0.473), but the performance of the radiomics model was inferior to that of the metabolism-based model.

It is common for patients with extra-nasal presentations to have more adverse clinical features (e.g., higher stage, poor performance status), and their survival rate is lower than for patients with nasal presentations (1, 49). Testicular ENKTCL is a highly aggressive malignancy, for which there is a paucity of established therapeutic regimens. Radical orchiectomy is commonly employed as the initial management strategy. Testicular ENKTCL may develop insidiously, most studies revealed survival rates of < 50% (50, 51). It is very common that patients with lesions restricted to the testis relapse within 6 months or die of widespread involvement of the skin, CNS, GI tract, and lungs within a year of the initial diagnosis (10). Kobayashi et al. (52) reported a case of testicular secondary ENKTCL in a 73-year-old man who was successfully treated with unilateral orchiectomy, DeVIC therapy, and radiotherapy to the contralateral testis. NK/T cell lymphoma patients are resistant to anthracycline-containing combination chemotherapy, with a 5-year survival rate of < 30% (23). Drénou et al. (53) suggested that this may be due to the development of multidrug resistance (MDR-1) genes. Yamaguchi et al. (54) concluded that radiotherapy of 50 Gy was required to achieve local control of ENKTCL. Unfortunately, however, radiation therapy alone is not sufficient to improve survival due to the significant number of patients who experience local and systemic recurrence after radiation therapy. The study by Ahn et al. (55) demonstrated that radiotherapy provided survival benefits to patients with only localized cutaneous involvements. The need for radiation therapy to the ipsilateral pelvis, para-aortic lymph nodes, and contralateral testes in patients with testicular ENKTCL is also uncertain. Although the occurrence of progression to the CNS frequently, prophylactic intrathecal chemotherapy to prevent CNS recurrence is often accompanied by considerable untoward effects (25). Clinical practice has recommended several combinations of radiotherapy and chemotherapy, such as concurrent, sequential, and sandwich chemoradiotherapy, as a therapeutic modality for localized ENKTCL with a low risk of treatment failure (56).

A multicenter-controlled study by Lee et al. (57) suggests that bone marrow or peripheral blood stem cell transplantation may be an effective treatment for ENKTCL. Studies of targeted drugs for ENKTCL are currently underway (58). EBV antigens might be targets for effector T cells based on the recent observation of the high efficacy of PD1 blockade (20). JAK/STAT, PDGF, aurora kinase, MYC, and NF-xB have been identified as potential therapeutic targets by Sanjay de Mei et al. (59). However, consensus on the standardized treatment of testicular ENKTCL has not been established, and in perspective, more comprehensive studies are necessary (60). In this study, we report two cases of primary testicular NK/T-cell lymphoma, one of which was characterized by bilateral nasal, left testicular, and right inguinal lymph node involvement following surgery, and the other associated with hemophagocytic lymphohistiocytosis. Both cases exhibited a dismal clinical course and prognosis.

## Conclusion

In summary, primary testicular ENKTCL is very rare clinically, mostly with a combined painful testicular mass as the first symptom, and is easily misdiagnosed as an inflammatory lesion. It has a highly aggressive clinical course, and we need to raise awareness of this disease and perform pathological histology, immunohistochemistry, and EBV-related tests as early as possible to clarify the diagnosis. ^18^F-FDG PET/CT plays pivotal roles in the diagnosis, staging, evaluation of treatment outcomes, and prognosis evaluation in patients with testicular ENKTCL, and it is helpful to assist clinical practice for better-individualized treatment plans. Indications for ^18^F-FDG PET/CT in this disease typically include: finding the location of the primary site in patients with elevated EBV DNA and tumor markers of unknown cause; to better clinically stage before treatment; monitoring treatment efficacy; and evaluating the situation of tumor recurrence and metastasis after treatment. To improve the early diagnosis, ^18^F-FDG PET/CT should be performed to accurately detect lesions that are not detected in conventional imaging methods and clarify the location and number of abnormal metabolic lesions, and guide the puncture site for pathological biopsy. No consensus about standardized treatment has been established, and a more comprehensive study is necessary.

## Data availability statement

The original contributions presented in the study are included in the article/supplementary material, further inquiries can be directed to the corresponding author.

## Ethics statement

This study was reviewed and approved by the Medical Ethics Committee of Peking University First Hospital. The patients and the minor's legal guardian provided their written informed consent to participate in this study. Written informed consent was obtained from the individual(s) and minor(s)' legal guardian for the publication of any potentially identifiable images or data included in this article. Written informed consent was obtained from the participant/patient(s) for the publication of this case report.

## Author contributions

WH: manuscript draft and editing. XL and LL: imaging data collection. YZ and YG: imaging data analysis. JG: supervision. LK: writing—review and editing. All authors met the requirements for authorship for the submitted version and agreed to its submission.
